# Comparative Analysis of Far East Sikhotinsky Rhododendron (*Rh. sichotense*) and East Siberian Rhododendron (*Rh. adamsii*) Using Supercritical CO_2_-Extraction and HPLC-ESI-MS/MS Spectrometry

**DOI:** 10.3390/molecules25173774

**Published:** 2020-08-19

**Authors:** Mayya Razgonova, Alexander Zakharenko, Sezai Ercisli, Vasily Grudev, Kirill Golokhvast

**Affiliations:** 1 N.I. Vavilov All-Russian Institute of Plant Genetic Resources, 190000 Saint-Petersburg, Russia; rarf@yandex.ru (A.Z.); k.golokhvast@vir.nw.ru (K.G.); 2 SEC Nanotechnology, Far Eastern Federal University, 690950 Vladivostok, Russia; 3 Agricultural Faculty, Department of Horticulture, Ataturk University, 25240 Erzurum, Turkey; sercisli@gmail.com; 4 Far Eastern Investment and Export Agency, 123112 Moscow, Russia; grudev@ya.ru; 5 Pacific Geographical Institute, Far Eastern Branch of the Russian Academy of Sciences, 690041 Vladivostok, Russia

**Keywords:** *Rhododendron sichotense*, Rhododendron adamsii, supercritical fluid extraction, HPLC–MS/MS, phenolic compounds

## Abstract

*Rhododendron sichotense* Pojark. and *Rhododendron adamsii* Rheder have been actively used in ethnomedicine in Mongolia, China and Buryatia (Russia) for centuries, as an antioxidant, immunomodulating, anti-inflammatory, vitality-restoring agent. These plants contain various phenolic compounds and fatty acids with valuable biological activity. Among green and selective extraction methods, supercritical carbon dioxide (SC-CO_2_) extraction has been shown to be the method of choice for the recovery of these naturally occurring compounds. Operative parameters and working conditions have been optimized by experimenting with different pressures (300–400 bar), temperatures (50–60 °C) and CO_2_ flow rates (50 mL/min) with 1% ethanol as co-solvent. The extraction time varied from 60 to 70 min. A HPLC-UV-VIS-ESI-MS/MS technique was applied to detect target analytes. A total of 48 different biologically active components have been identified in the *Rh. adamsii* SC-CO_2_ extracts. A total of 31 different biologically active components have been identified in the *Rh. sichotense* SC-CO_2_ extracts.

## 1. Introduction

A total of 19 species of rhododendrons are growing in the territory of Russia, the main part of which (13 species) are found only in the flora of the Russian Far East and Eastern Siberia [1]. Russian researchers classify the genus *Rhododendron* somewhat differently than foreign ones [2]. At present, there is no unified classification scheme for a taxon, since the genus is very large—more than 800 species, as well as the presence of a large number of convergent characters among its representatives, complicating the construction of a natural classification [3].

The genus system, developed and adopted by Russian scientists, divides the genus *Rhododendron* into subgenera and series. In it, *Rhododendron adamsii* Rehder and *Rhododendron parvifolium* Adams are assigned to the subgenus *Osmothamnus* Maximowicz (*Fragrantia* E. Busch series and *Parvifolia* E. Busch series) (Table 1). Species of *Rh. dauricum* L., *Rh. ledebourii* Pojarkova, *Rh. sichotense* Pojarkova and *Rh. micronulatum* Turczaninowia constitute the series *Daurica* Pojarkova subgenus *Rhodorastrum* (Maxim.) Drude [4].

*Rhododendron sichotense* Pojark. is a plant from the genus of rhododendrons [5]. It grows in the Far East (Primorsky Krai) on the eastern slope of the Sikhote Alin ridge. This type of rhododendron is included in the Red Book of the Russian Federation [6]. From its closest relatives, *Rhododendron dauricum* and *Rhododendron micronulatum* is distinguished by larger, sometimes more than 7 cm wide, flowers and wider, green leaves from the underside, not falling leaves. Large flowers, lush foliage, winter hardiness suggest that *Rh. sichotense* is a very promising garden plant for areas with a harsh climate [7].

*Rh. adamsii* Rheder is a shrub found in Eastern Siberia and Baikal in the alpine and subalpine zones of the mountains, forming a shrub tundra, and at the upper border of the forest at an altitude of 1200–2500 m above sea level. This is a shrub from 1 to 3 m in height with falling leaves. The flowers are pink, large enough, and open before the leaves appear. The leaves are narrow, green above, grayish green below, and very fragrant. *Rh. adamsii* grows in pine and deciduous forests with grass and moss, on forest edges and especially on rocky mountains [8].

Traditional medicine uses different types of rhododendron to treat a number of diseases of the respiratory system, the gastrointestinal tract, chronic skin diseases, hypertension, rheumatism, helminthiases, etc. [9]. The stupefying smell formed during the burning of leaves and caused by the sharp evaporation of volatile terpenes has long been used by indigenous ethnic groups of Siberia and Far East as a psychoactive, analgesic, and narcotic drug [10,11].

*Rh. adamsii* Rehder is used as a stimulant and tonic by the populations of Buryatia, Mongolia and China. Decoctions and tinctures of it are used for cold diseases, as a diuretic agent for cardiac edema, as well as an adaptogen [12]. Pharmacological studies have shown that *Rh. adamsii* has antimicrobial, anti-inflammatory, immunomodulating, antioxidant effects [13,14].

In essential oil of *Rh. adamsii*, it is possible to isolate the components present both in the leaves and stems of the plant: α- and β-pinenes, β-myrcene, cis-β-ocimene, isoledene, aromadendrene, humulene, β-farnesol, γ-murolene, β-selinene, ledene, α-farnesol, δ-cadinene, trans-nerolidol, spathulenol, β-elemenone, germacrone [15]. The essential oil of the stems of the plant contains germacrene D and germacrene B, which are absent from the essential oil of the leaves. In almost all samples of essential oil of leaves and stems of *Rh. adamsii* there is found 4-phenyl-2-butanone, the content of which is from 3 to 13%, as well as its related 4-phenyl-2-butanol, the content of which is from 1.9 to 7.4% [16].

This study considers the possibility and effectiveness of supercritical carbon dioxide (SC-CO_2_) extraction of biologically active substances from stems and leaves of Rh. adamsii. Previously, the authors of this article successfully used SC-CO_2_ extraction to obtain biologically active substances from plants of the Far Eastern taiga *Panax ginseng*, *Rhodiola rosea*, and *Schisandra chinensis*, which are extremely popular in traditional medicines of Southeast Asia [17,18].

Supercritical fluid extraction (SFE) has been used since the late 1970s to analyze food products, isolate biologically active substances and determine lipid levels in food, as well as levels of toxic substances [19,20,21]. The use of SFE for fractionation and/or enrichment of certain components in products has been reported since the 1980s [22,23,24]. In addition, the products do not have residues of organic solvents, which occur with conventional extraction methods, and solvents can be toxic, for example, in the case of methanol and *n*-hexane. Easy solvent removal from the final product, high selectivity and the use of moderate temperatures in the extraction process are the main attractive factors of supercritical technology, leading to a significant increase in research for use in the food and pharmaceutical industries [25,26,27].

An alternative to the use of co-solvents in the case of poorly soluble or practically insoluble compounds is to completely change the process scheme using the so-called supercritical solvent extraction (SAE). Industrial-scale devices with technological schemes containing CO_2_ processing plants have already been developed; therefore, most of the solvent/anti-solvent is recovered. SAE increases are associated with the same process conditions as the pressure, temperature and concentration of solutes in the slurry. However, the main parameter is the molar fraction of CO_2_. It depends on the relative flow rate of the CO_2_ and the solvent liquid to set the supercritical precipitant composition for the CO_2_/solvent mixture used [28].

Popova et al. [29] (2018) investigated the possibility of SC-CO_2_ extracting chlorophylls and carotenoids of *Ledum palustre* L. (*Rhododendron tomentosum* Harmaja) by supercritical fluid extraction using supercritical carbon dioxide and a co-solvent of ethyl alcohol as a solvent. It has been found that by varying the pressure and temperature of the fluid, the duration of processing and the moisture content of the raw material, extracts can be obtained enriched in one or both of the recoverable pigments. Furthermore, in this case, the amount of ethanol used as a co-solvent was 5% and was necessary and sufficient for efficient extraction of pigments with supercritical CO_2_.

The anti-inflammatory activity of two extracts from the aerial parts of *Ledum palustre* L. (*Rh. tomentosum* Harmaja) has been reported by [30]. The volatile oil was obtained by SC-CO_2_ extraction and the essential oil by hydrodistillation (HD). The anti-inflammatory activity was evaluated by the subcutaneous carrageenan injection-induced hind paw oedema. The results show that *L. palustre* essential oil enhanced a significant inhibition of oedema (50–73%) for HD oil and (52–80%) for SFE oil.

The results of SC-CO_2_-extraction of leaves and branches of rhododendrons, in particular, indicated that, when using this technology, the extract contained all biologically active components of the plant, as well as inert mixtures of extracted compositions. This study is devoted to comparative mass spectrometry of extracted biologically active substances from two closely related subgenus of rhododendron: *Rh. sichotense* Pojark. and *Rh. adamsii* Rehder.

## 2. Results and Discussion

In the first instance, the influence of the supercritical parameters (CO_2_ flow rate, temperature, % co-solvent, and pressure) on extraction yield was investigated. The cumulative quantitative extracts yield are summarized in the Table 2. Several experimental conditions were investigated working in a pressure range of 300–400 bar, with co-solvent EtOH and a temperature range of 50–60 °C.

**Figure 1:**
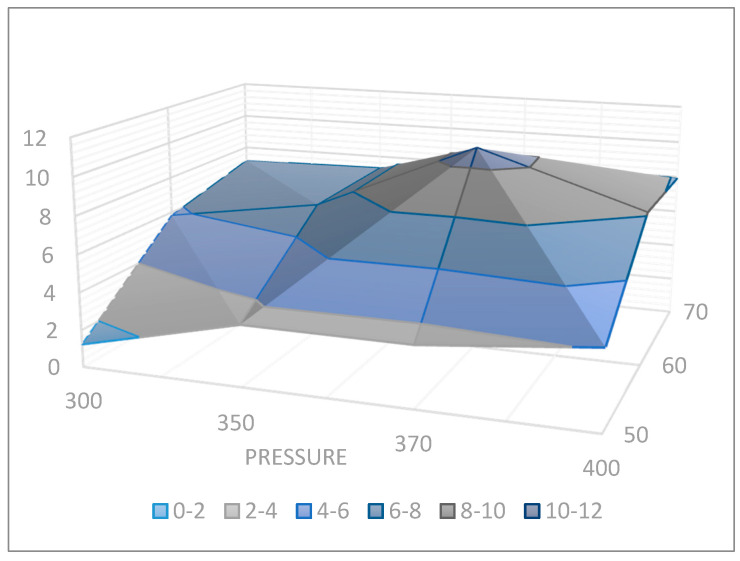
Orthogonal projection representing the extraction yield of target analytes at 300 to 400 Bar and 50–70 °C.

Orthogonal projection representing the extraction yield at 300 to 400 Bar and 50–70 °C is shown in Figure 1. The best results were obtained at 370 Bar and 60 °C. An ion trap amaZon SL BRUKER DALTONIKS equipped with an electron spray ionization (ESI) source in the negative and positive ion modes and analysis of fragmented ions was used in this scientific work.

A screening of biologically active substances from *Rh. adamsii* sample and *Rh. sichotense* sample was obtained using this method. Typical base peak chromatograms (BPC) of analyzed target analytes are shown in the Supplementary Material. Identification of compounds was assigned by comparison of their UV-Vis spectra and mass spectrometric data obtained under both negative and positive electron spray ionization (ESI^−^/ESI^+^) conditions and with the scientific literature. Under these conditions a total of 800 peaks were detected in the ion chromatogram.

Series studies by HPLC–MS/MS of both samples of rhododendrons (*Rh. sichotense* and *Rh. adamsii)* were carried out, and the results of studies of the target compounds are presented below (Table 3).

Table 4 summarized all the molecular masses of the target analytes isolated from SC-CO_2_ of *Rh. sichotense* and *Rh. adamsii.* Among them, 57 biologically active substances were authenticated (*m*/*z* values and fragment ions) by comparison with the literature data [4,5,10,11,12,13,15,16,30,31,32,33,34,35,36,37,38,39,40,41,42,43,44,45,46,47,48,49,50,51,52,53]. A total of 48 different biologically active components have been identified in the *Rh. adamsii* SC-CO_2_ extracts. A total of 31 different biologically active components have been identified in the *Rh. sichotense* SC-CO_2_ extracts.

**Figure 2:**
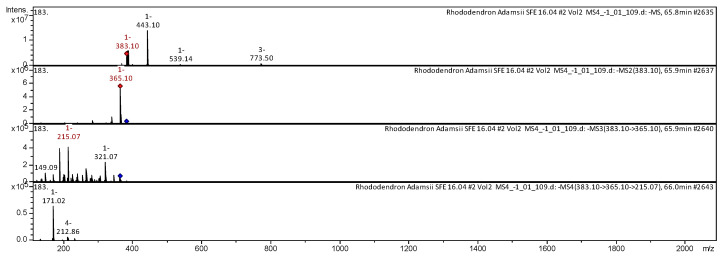
Collision-induced dissociation (CID) spectrum of fraxetin-7-*O*-beta-glucoronide from *Rh. adamsii*, *m*/*z* 383.10.

**Figure 3:**
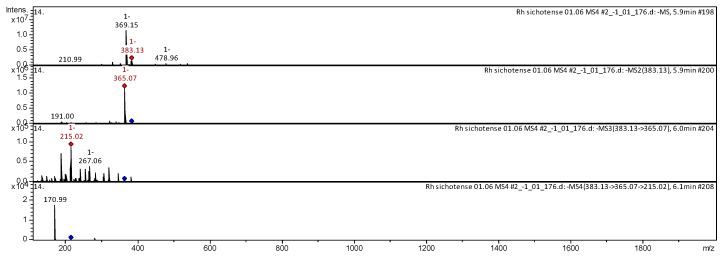
CID spectrum of fraxetin-7-*O*-beta-glucoronide from *Rh. sichotense*, *m*/*z* 383.13.

**Figure 4:**
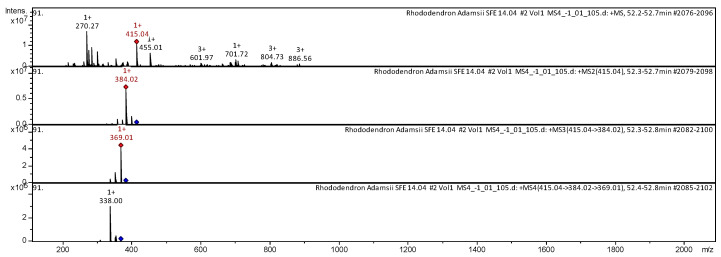
CID spectrum of Beta-sitosterin from *Rh. adamsii*, *m*/*z* 415.04.

**Figure 5:**
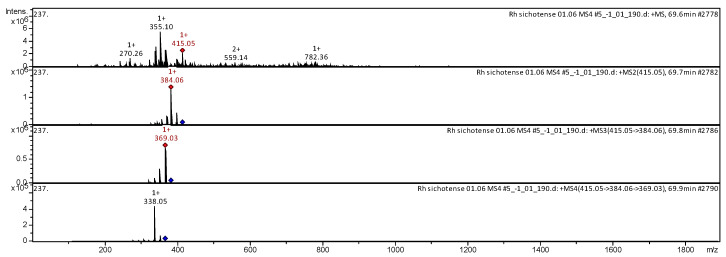
CID spectrum of Beta-sitosterin from *Rh. sichotense*, *m*/*z* 415.05.

**Figure 6:**
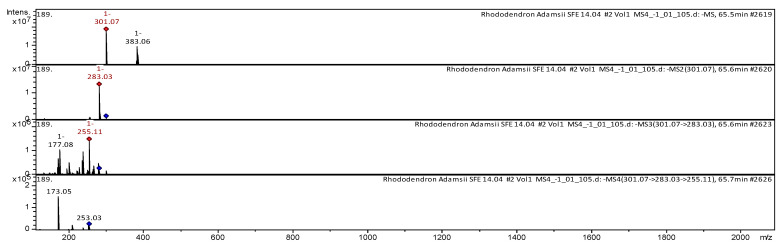
CID spectrum of quercetin from *Rh. adamsii*, *m*/*z* 301.07.

**Figure 7:**
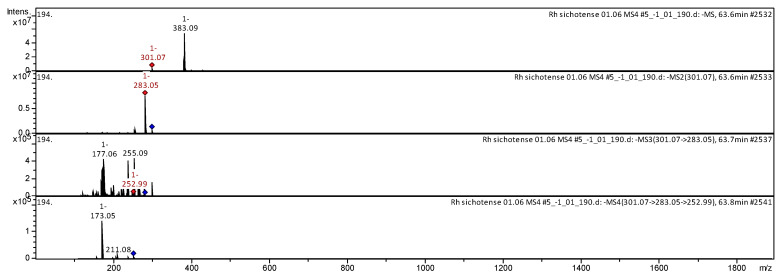
CID spectrum of quercetin from *Rh. sichotense*, *m*/*z* 301.07.

**Figure 8:**
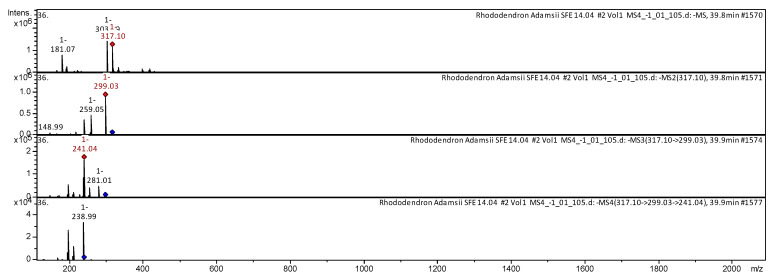
CID spectrum of myricetin from *Rh. adamsii*, *m*/*z* 317.10.

**Figure 9:**
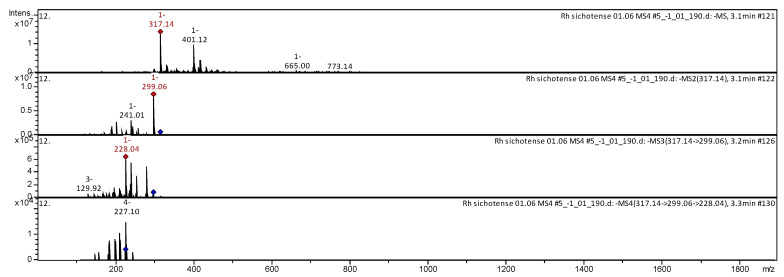
CID spectrum of myricetin from *Rh. sichotense*, *m*/*z* 317.14.

**Figure 10:**
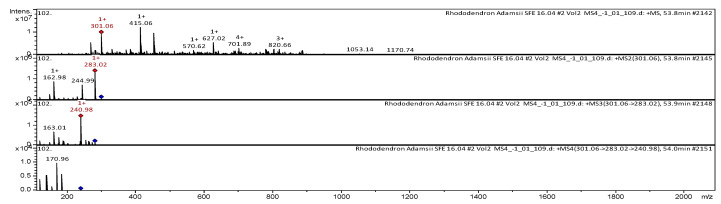
CID spectrum of farrerol from *Rh. adamsii*, *m*/*z* 301.06.

**Figure 11:**
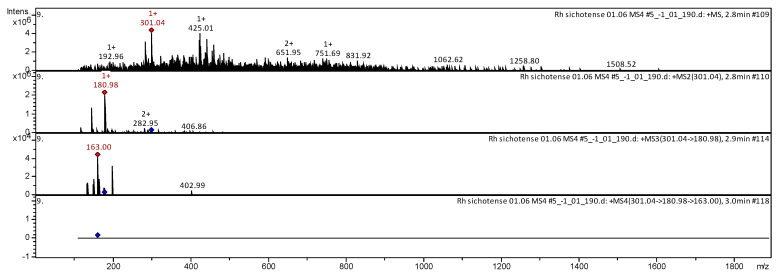
CID spectrum of farrerol from *Rh. sichotense*, *m*/*z* 301.06.

Figure 2, Figure 3, Figure 4, Figure 5, Figure 6, Figure 7, Figure 8, Figure 9, Figure 10 and Figure 11 shows examples of the decoding spectra (collision-induced dissociation (CID) spectrum) of the ion chromatogram obtained using tandem mass spectrometry. The CID spectrum in negative ion modes of fraxetin-7-*O*-beta-glucoronide from *Rh. adamsii* and *Rh. sichotense* are shown in Figure 2 and Figure 3.

The [M − H]^−^ ion produced fragment ion with *m*/*z* 383.10 (Figure 2). The fragment ion with *m*/*z* 383.10 produced characteristic daughter ion with *m*/*z* 321.07, *m*/*z* 215.07 and *m*/*z* 149.09. The fragment ion with *m*/*z* 215.07 formed two daughter ions with *m*/*z* 171.02, *m*/*z* 212.86. It was identified in the bibliography in extract from rhododendron *L. palustre* [13,30,31,32,33,45].

The [M − H]^−^ ion produced fragment ion with *m*/*z* 383.13 (Figure 3).The fragment ion with *m*/*z* 383.13 produced two fragments with *m*/*z* 365.07, *m*/*z* 191.00. The fragment ion with *m*/*z* 365.07 produced two characteristic daughter ions with *m*/*z* 267.06 and *m*/*z* 215.02. The fragment ion with *m*/*z* 215.02 formed a daughter ion with *m*/*z* 170.99. It was identified in the bibliography of the methanolic extract from rhododendron *L. palustre* [30,31,32,33,44].

The CID spectrum in positive ion modes of Beta-sitosterin from *Rh. adamsii* and *Rh. sichotense* is shown in Figure 4 and Figure 5. 

The [M + H]^+^ ion produced one fragment with *m*/*z* 415.04 (Figure 4). The fragment ion with *m*/*z* 384.02 produced one daughter ion with *m*/*z* 369.01. The fragment ion with *m*/*z* 369.01 formed a daughter ion with *m*/*z* 338.00. It was identified in the bibliography in the extract from rhododendrons *L. palustre* [30,31,32,33,41,44,45] and *Rh. adamsii* [10,12,15,16,42,52].

The [M + H]^+^ ion produced one fragment with *m*/*z* 384.06 (Figure 5). The fragment ion with *m*/*z* 384.06 produced one daughter ion with *m*/*z* 369.03. The fragment ion with *m*/*z* 369.03 formed a daughter ion with *m*/*z* 338.05. It was identified in the bibliography in the extract from rhododendrons *L. palustre* [30,31,32,33,41,44,45] and *Rh. adamsii* [10,12,15,16,42,52].

The CID spectrum in negative ion modes of quercetin from *Rh. adamsii* and *Rh. sichotense* is shown in Figure 6 and Figure 7.

The [M − H]^−^ ion produced one fragment ion with *m*/*z* 283.03 (Figure 6). The fragment ion with *m*/*z* 283.03 produced two daughter ions with *m*/*z* 255.11 and *m*/*z* 177.08. The fragment ion with *m*/*z* 255.11 formed two daughter ions with *m*/*z* 253.03 and *m*/*z* 173.05. It was identified in the bibliography in extracts from rhododendrons *Rh. sichotense, Rh. micronulatum* [38,39,40]; *Rh. ungernii* [34]; *Rhodiola crenulata* [36]; *Rh. adamsii* [10,12,15,16,42,52]; *Rh. parvifolium* [10]; *Ocimum* [43].

The [M − H]^−^ ion produced one fragment ion with *m*/*z* 283.03 (Figure 7). The fragment ion with *m*/*z* 283.03 produced three daughter ions with *m*/*z* 255.09, *m*/*z* 177.06 and *m*/*z* 252.99. The fragment ion with *m*/*z* 252.99 formed two daughter ions with *m*/*z* 211.08 and *m*/*z* 173.05. It was identified in the bibliography in extracts from rhododendrons *Rh. sichotense, Rh. micronulatum* [38,39,40]; *Rh. ungernii* [34]; *Rhodiola crenulata* [36]; *Rh. adamsii* [10,12,15,16,42,52]; *Rh. parvifolium* [10]; *Ocimum* [43]. 

The CID spectrum in negative ion modes of myricetin from *Rh. adamsii* and *Rh. sichotense* is shown in Figure 8 and Figure 9. 

The [M − H]^−^ ion produced three fragment ions with *m*/*z* 299.03, *m*/*z* 259.05 and *m*/*z* 148.99 (Figure 8). The fragment ion with *m*/*z* 299.03 produced two daughter ions with *m*/*z* 241.04 and *m*/*z* 281.01. The fragment ion with *m*/*z* 241.04 formed a daughter ion with *m*/*z* 238.99. It was identified in the bibliography in extracts from rhododendrons *Rh. sichotense, Rh. micronulatum* [39,40,41]; *Rh. ungernii* [35]; *Rhodiola crenulata* [37]; *Rh. adamsii* [16,17,43,53]; *Rh. parvifolium* [41]; *Ocimum* [44]. 

The [M − H]^−^ ion produced two fragment ions with *m*/*z* 299.06, *m*/*z* 241.01 (Figure 9). The fragment ion with *m*/*z* 299.06 produced two daughter ions with *m*/*z* 228.04 and *m*/*z* 129.92. The fragment ion with *m*/*z* 228.04 formed a daughter ion with *m*/*z* 227.10. It was identified in the bibliography in extracts from rhododendrons *Rh. sichotense* [4,5]; *Rh. ungernii* [34]; *Rhodiola crenulata* [36]; *Rh. adamsii* [10,15,16,42,52]; *Rh. parvifolium* [10,40].

The CID spectrum in positive ion modes of farrerol from *Rh. adamsii* and *Rh. sichotense* is shown in Figure 10 and Figure 11. The [M + H]^+^ ion produced three fragment ions with *m*/*z* 283.02, *m*/*z* 244.99 and *m*/*z* 162.98 (Figure 10). The fragment ion with *m*/*z* 283.02 produced two daughter ions with *m*/*z* 240.98 and *m*/*z* 163.01. The fragment ion with *m*/*z* 240.98 formed a daughter ion with *m*/*z* 170.96. It was identified in the bibliography in extracts from rhododendrons *Rh. dauricum* [38,39,40,41]; *Rh. ungernii* [34].

The [M + H]^+^ ion produced two fragment ions with *m*/*z* 282.95 and *m*/*z* 180.98 (Figure 11). 

The fragment ion with *m*/*z* 180.98 formed a daughter ion with *m*/*z* 163.00. It was identified in the bibliography in extracts from rhododendrons *Rh. dauricum* [38,39,40]; *Rh. ungernii* [34].

## 3. Materials and Methods

### 3.1. Materials

The objects of study were purchased samples of *Rh. sichotense* (leaves and stems) from Primorsky Krai (the eastern slope of the Sikhote Alin ridge) and *Rh. adamsii* (leaves and stems) from the area near lake Baykal, Russia. All samples were morphologically authenticated according to the current standard of Russian Pharmacopeia [54]. All samples were immediately washed weighed by 10 g aliquot then frozen and kept until extraction.

### 3.2. Chemicals and Reagents

HPLC-grade acetonitrile was purchased from Fisher Scientific (Southborough, UK), MS-grade formic acid was from Sigma-Aldrich (Steinheim, Germany). Ultra-pure water was prepared from a SIEMENS ULTRA clear (SIEMENS water technologies, Munich, Germany), and all other chemicals were analytical grade.

### 3.3. Liquid Chromatography

HPLC was performed using a Shimadzu LC-20 Prominence HPLC (Kanda-Nishikicho 1-chrome, Shimadzu, Chiyoda-ku, Tokyo, Japan), equipped with a UV-sensor and a Shodex ODP-40 4E (250 × 4.6 mm, particle size: 4 μm) reverse phase C18 column to perform the separation of multicomponent mixtures. The gradient elution program was as follows: 0.01–4 min, 100% A; 4–60 min, 100–25% A; 60–75 min, 25–0% A; control washing 75–120 min 0% A. The entire HPLC analysis was performed using a UV-VIS detector SPD-20A (Kanda-Nishikicho 1-chrome, Shimadzu, Chiyoda-ku, Tokyo, Japan) at wavelengths of 230 and 330 nm, at 17 °C provided with a column oven CTO-20A (Kanda-Nishikicho 1-chrome, Shimadzu, Chiyoda-ku, Tokyo, Japan) with an injection volume of 20 μL.

### 3.4. SC-CO_2_ Extraction

SC-CO_2_ extraction was performed using the Supercritical fluid system -500 (Thar SCF Waters, Milford, MA, USA) supercritical pressure extraction apparatus. System options include: Co-solvent pump (Thar Waters P-50 High Pressure Pump), for extracting polar samples. CO_2_ flow meter (Siemens, Munich, Germany), to measure the amount of CO_2_ being supplied to the system, multiple extraction vessels, to extract different sample sizes or to increase the throughput of the system. Flow rate was 50 mL/min for liquid CO_2_ and 1.00 mL/min for EtOH. Samples for extraction of 10 g of frozen *Rh. sichotense* and *Rh. adamsii* pre-cut into pieces no more than 1 cm were used. Several experimental conditions were investigated, working in a pressure range of 300–400 bar, with 1% of C_2_H_5_OH as co-solvent and a temperature range of 50–60 °C. The extraction time was counted after reaching the working pressure and equilibrium flow, and it was 60–70 min for each sample.

### 3.5. Mass Spectrometry

MS analysis was performed on an ion trap amaZon SL (BRUKER DALTONIKS, Bremen, Germany) equipped with an ESI source in negative ion mode. The optimized parameters were obtained as follows: ionization source temperature: 70 °C, gas flow: 4 L/min, nebulizer gas (atomizer): 7.3 psi, capillary voltage: 4500 V, end plate bend voltage: 1500 V, fragmentary: 280 V, collision energy: 60 eV MS/MS MS^4^ (four stages of separation). An ion trap was used in the scan range *m*/*z* 100–1700 for MS and the capture rate was one spectrum/s for MS and two spectrum/s for MS/MS. All experiments were repeated three times. A four-stage ion separation mode (MS/MS mode) was implemented.

## 4. Conclusions

Aiming to optimize the extraction of target analytes from the *Rh. sichotense* and *Rh. adamsii* leaves and stems, several experimental conditions were investigated working in a pressure range of 300–400 bar, with 1% of C_2_H_5_OH as co-solvent and a temperature range of 50–60 °C. Although this approach is not quantitative for evaluation of each analyte, it is semiquantitative when comparing a series of extractions and allows better comparison of the yield without loss of individual analytes during fractionation and sample preparation. The best results were obtained at 370 bar and 60 °C.

High-accuracy mass spectrometric data were recorded on an ion trap amaZon SL BRUKER DALTONIKS equipped with an ESI source in the negative ion mode. The four-stage ion separation mode was implemented to perform correct identification. Under these conditions a total of 800 peaks were detected in the ion chromatogram. An optimized extraction process with SC-CO_2_ (co-solvent 1% ethanol) provided the samples for an accurate analytical study by HPLC–MS/MS technique. A total of 50 different biologically active components were identified in the *Rh. adamsii* SC-CO_2_ extracts. A total of 30 different biologically active components were identified in the *Rh. sichotense* SC-CO_2_ extracts.

An analysis of the similarity of the composition of biologically active components of *Rh. sichotense* and *Rh. adamsii* revealed their significant relationship. An analysis of morphometric parameters and composition of *Rh. sichotense* and *Rh. adamsii* flavonoids from populations of the Far East and Siberia indicated the presence of ecological and geographical variability. Inter-population differences in morphometric indicators are more significant than in chemical ones. Moreover, the former is more associated with climatic factors, while the latter are more associated with edaphic growth factors. The *Rh. adamsii* extract was more diverse in chemical composition than *Rh. sichotense*. A particularly large difference was observed in acidic components such as caffeic acid, azelaic acid, myristic acid, pentadecanoic acid, palmitoleic acid, linoleic acid. The extract of *Rh. sichotense* contained mainly stearic acid, nonadecanoic acid, montanic acid, behenic acid, tetracosanoic acid and chlorogenic acid. The flavonoid content of both rhododendrons was mainly the same.

These data could support future research for the production of a variety of pharmaceutical products containing ultra-pure SC-CO_2_ extracts of *Rh. sichotense* and *Rh. adamsii*. The richness of various biologically active compounds, including flavonoids: quercetin, kaempferol, dihydroquercetin, farrerol, myricetin, etc. provides great opportunities for the design of new drugs based on extracts from this species of rhododendron.

## Figures and Tables

**Table 1:** Classification of genus *Rhododendron* L.

No.	Species or Variety	Subgenus	Row
1	*Rh. sichotense* Pojark.; *Rh. micronulatum* Pojark.; *Rh. dauricum* L.; *Rh. ledebourii* Pojark.	*Rhodorastrum*	Daurica Pojark.
2	*Rh. parvifolium* Adams [*Rh. lapponicum* (L.) Wahlenb.]	*Osmothamnus* Maximowicz	*Parvifolia* E. Busch
3	*Rh. adamsii* Rehd. [*Rh. fragrans* (Adams) Maxim.]		*Fragrantia* E. Busch

**Table 2:** Extraction yield of *Rh. adamsii* presented depending on operational parameters (pressure, temperature, CO2 flow rate, % co-solvent).

No.	Temperature (°C)	Pressure (Bar)	CO_2_ Flow Rate (mL/min)	% Co-Solvent EtOH	Extraction Yield (mg/g)
1	50	300	30	1	1.23
2	50	350	50	2	3.27
3	50	370	30	1	3.25
4	50	400	50	1	4.15
5	60	300	30	2	5.81
6	60	350	30	2	7.12
7	60	370	50	1	10.86
8	60	400	30	2	8.13
9	70	300	30	1	7.15
10	70	350	50	1	7.28
11	70	370	30	1	8.10
12	70	400	30	1	7.90

**Table 3:** Compounds identified from SC-CO_2_ extracts by two varieties of rhododendron: *Rh. sichotense* and *Rh. adamsii.*

NO.	Identification	Formula	Calculated Mass	*Rh. adamsii* (104)	*Rh. adamsii* (105)	*Rh. adamsii* (109)	*Rh. adamsii* (110)	*Rh. adamsii* (108)	*Rh. adamsii* (112)	*Rh. adamsii* (116)	*Rh. adamsii* (117)	*Rh. adamsii* (118)	*Rh. adamsii* (122)	*Rh. adamsii* (123)	*Rh. sicho* (175)	*Rh. sicho* (176)	*Rh. sicho* (177)	*Rh. sicho* (178)	*Rh. sicho* (190)	*Rh. sicho* (192)	*Rh. sicho* (193)	References
1	Lepalol [5-(3-Furyl)-2-methyl-1-penten-3-ol	C_10_H_14_O_2_	166.217																			[31,45,48]
2	Caffeic acid [(2E)-3-(3,4-Dihydroxyphenyl)acrylic acid]	C_9_H_8_O_4_	180.1574																			[10,35,42,43,50]
3	Azelaic acid (Nonanedioic acid)	C_9_H_16_O_4_	188.2209																			[15,16]
4	Calamenene [Cis-Calamenene]	C_15_H_22_	202.3352																			[15,16,38]
5	Germacron	C_15_H_22_O	218.3346																			[4,5,10,42,44]
6	Myristic acid (Tetradecanoic acid; *N*-Tetradecanoic acid)	C_14_H_28_O_2_	228.3709																			[15,16]
7	Pentadecanoic acid (Pentadecylic acid)	C_15_H_30_O_2_	242.3975																			[15,16]
8	Palmitoleic acid	C_16_H_30_O_2_	254.4082																			[15,16]
9	Cis-cyclopropan-9,10-hexadecanoic acid	C_17_H_32_O_2_	268.4348																			[15,16]
10	Linoleic acid (Linolic acid; Telfairic acid)	C_18_H_32_O_2_	280.4455																			[15,16,49]
11	Stearic acid (Octadecanoic acid; Stearophanic acid)	C_18_H_36_O_2_	284.4772																			[15,16]
12	Kaempferol	C_15_H_10_O_6_	286.2363																			[36,39,40,43,50]
13	Cis-cyclopropan-9,10-octadecanoic acid	C_19_H_32_O_2_	292.4562																			[15,16]
14	Nonadecanoic acid (*N*-Nonadecanoic acid)	C_19_H_38_O_2_	298.5038																			[15,16]
15	Kaempferol 5-methyl ether	C_16_H_12_O_6_	300.2629																			[39]
16	Farrerol	C_17_H_16_O_5_	300.3059																			[34,39]
17	Quercetin	C_15_H_10_O_7_	302.2357																			[34,36,39,40,43,50]
18	Dihydroquercetin (Taxifolin; Taxifoliol)	C_15_H_12_O_7_	304.2516																			[35,39,40]
19	Cannabigerorcinic acid (Cannabigerorcinolic acid; Cannabiorcogerolic acid	C_18_H_24_O_4_	304.3808																			[15,16]
20	Docosane	C_22_H_46_	310.6006																			[48]
21	8-Demethyleucalyptin [5-Hydroxy-4′,7-dimetoxy-6-methylflavone; Pabalate; Sodium salicylate]	C_18_H_16_O_5_	312.3166																			[33]
22	Arachic acid (Arachidic acid; eicosanoic acid)	C_20_H_40_O_2_	312.5304																			[15,16]
23	Azaleatin [5-*O*-Methylquercetin]	C_16_H_12_O_7_	316.2623																			[10,39,41,42]
24	Myricetin	C_15_H_10_O_8_	318.2351																			[36,34,39,40,50]
25	Gossypetin [Articulatidin; Equisporol]	C_15_H_10_O_8_	318.2351																			[37]
26	Ampelopsin [Dihydromyricetin; Ampeloptin]	C_15_H_12_O_8_	320.251																			[39]
27	Heneicosanoic acid (Heneicosylic acid)	C_21_H_42_O_2_	326.557																			[15,16]
28	Myricetin 5-Methyl ether [5-*O*-Methylmyricetin]	C_16_H_12_O_8_	332.2617																			[39]
29	Esculin [Aesculin; Esculoside; Polichrome]	C_15_H_16_O_9_	340.2821																			[33,41]
30	Behenic acid (Docosanoic acid)	C_22_H_44_O_2_	340.5836																			[15,16]
31	Pentacosane (*N*-Pentacosane)	C_25_H_52_	352.6854																			[48]
32	Chlorogenic acid	C_16_H_18_O_9_	354.3087																			[10,35,42]
33	Scopolin [Scopoloside; Scopoletin-7-glucoside; Murrayin]	C_16_H_18_O_9_	354.3087																			[41]
34	Tricosanoic acid (*N*-Tricosanoic acid)	C_23_H_46_O_2_	354.6101																			[15,16]
35	Lignoceric acid (Tetracosanoic acid)	C_24_H_48_O_2_	368.6367																			[15,16]
36	Fraxin (Fraxetin-8-*O*-glucoside)	C_16_H_18_O_10_	370.3081																			[33]
37	Daurichromenic acid	C_23_H_30_O_4_	370.4819																			[15,16]
38	Pentacosanoic acid (*N*-Pentacosanoic acid)	C_25_H_50_O_2_	382.6633																			[15,16]
39	Fraxetin-7-*O*-beta-glucuronide	C_16_H_16_O_11_	384.2916																			[41]
40	Beta-Sitosterin [Beta-Sitosterol]	C_29_H_50_O	414.7067																			[10,30,42]
41	Cyanidin-3-alpfa-l-arabinoside	C_20_H_19_O_10_	419.3589																			[10,42]
42	Montanic acid (Amyrin; Beta-Amyrenol)	C_28_H_56_O_2_	424.743																			[15,16]
43	Alpha-Amyrin [Viminalol]	C_30_H_50_O	426.7174																			[30]
44	Lupeol [Fagarasterol; Clerodol; Monogynol B; Lupenol]	C_30_H_50_O	426.7174																			[30]
45	Dihydroquercetin-3-arabinofuranoside	C_20_H_16_O_11_	432.3344																			[10,42]
46	Afzelin [ Kaempferol-3-Rhamnoside; Kaempferin]	C_21_H_20_O_10_	432.3775																			[39,40]
47	Quercetin-3-*O*-beta-xyloside (Reynoutrin; Quercetin 3-*O*-Beta-d-Xylopyranoside)	C_20_H_17_O_11_	433.3424																			[34]
48	Avicularin (Quercetin 3-Alpha-l-Arabinofuranoside; Avicularoside)	C_20_H_18_O_11_	434.3503																			[10,33,39,40,42]
49	Pentoside dihydroquercetin		436																			[40]
50	Erithrodiol [3-beta-Erytrodiol]	C_30_H_50_O_2_	442.7168																			[30]
51	Uvaol	C_30_H_50_O_2_	442.7168																			[30]
52	Quercitrin [Quercetin 3-l- Rhamnoside; Quercetrin]	C_21_H_20_O_11_	448.3769																			[33,39,46]
53	Catechin-7-*O*-glucoside	C_21_H_24_O_11_	452.4087																			[34]
54	Micromeric acid	C_30_H_46_O_3_	454.6844																			[30]
55	Hyperoside (Quercetin 3-*O*-galactoside; Hyperin)	C_21_H_20_O_12_	464.3763																			[10,33,34,39,40,41,42]
56	Quercetin 3-*O*-glucoside [ Isoquercitrin]	C_21_H_20_O_12_	464.3763																			[33,46]
57	Alpha.-Tocopherol-Beta-d-Mannoside [Dihydro-2H-Chromen-6-YI Hexofuranoside]	C_35_H_60_O_7_	592.8467																			[48]

Colors are added for readability to avoid confusing columns. Green shades *Rh. adamsii*. blue *Rh. sichotense*.

**Table 4:** Components identified from the SC-CO_2_ extracts of *Rh. sichotense* and *Rh. adamsii*.

No.	Identification	Formula	Calculated Mass	Observed Mass [M − H]^−^	Observed Mass [M + H]^+^	Observed Mass [M + Na]^+^	MS/MS Stage 2 Fragmentation	MS/MS Stage 3 Fragmentation	MS/MS Stage 4 Fragmentation	Species of Rhododendron
1	Lepalol [5-(3-Furyl)-2-methyl-1-penten-3-ol	C_10_H_14_O_2_	166.217	165.06			147.01			*Rh. adamsii*
2	Caffeic acid [(2E)-3-(3,4-Dihydroxyphenyl) acrylic acid]	C_9_H_8_O_4_	180.1574		181.08		163.03; 135.11			*Rh. adamsii*
3	Azelaic acid [Nonanedioic acid]	C_9_H_16_O_4_	188.2209			210.09	192.12	175.06; 136.12		*Rh. adamsii*
4	Calamenene [Cis-Calamenene]	C_15_H_22_	202.3352		203.09		147.05	119.06		*Rh. sichotense*
5	Germacron	C_15_H_22_O	218.3346		219.06		201.07; 149.07	159.07		*Rh. adamsii*
6	Myristic acid (Tetradecanoic acid; *N*-Tetradecanoic acid)	C_14_H_28_O_2_	228.3709			251.09	150.48	149.08		*Rh. adamsii*
7	Pentadecanoic acid (Pentadecylic acid)	C_15_H_30_O_2_	242.3975		243.06		201.01; 137.05	181.05; 135.04		*Rh. adamsii*
8	Palmitoleic acid	C_16_H_30_O_2_	254.4082			277.09	275.04; 207.05	256.99	157.11	*Rh. adamsii*
9	Cis-cyclopropan-9,10-hexadecanoic acid	C_17_H_32_O_2_	268.4348		269.02		185.97; 121.08	176.96	154.98	*Rh. adamsii*
10	Linoleic acid [Linolic acid; Telfairic acid]	C_18_H_32_O_2_	280.4455			303.06	285.05; 163.00	180.95; 135.06	162.99	*Rh. adamsii*
11	Stearic acid [Octadecanoic acid; Stearophanic acid]	C_18_H_36_O_2_	284.4772		285.07		284.18; 229.07; 163.02	180.90; 135.05	163.03	*Rh. adamsii; Rh. sichotense*
12	Kaempferol [3,5,7-Trihydroxy-2-(4-hydro- xyphenyl)-4-*H*-chromen-4-one]	C_15_H_10_O_6_	286.2363		287.00		286.24; 204.96; 163.02	181.02	162.88	*Rh. adamsii*
13	Cis-cyclopropan-9,10-octadecanoic acid	C_19_H_32_O_2_	292.4562		293.05		274.98	256.99; 162.98	201.03	*Rh. adamsii*
14	Nonadecanoic acid [*N*-Nonadecanoic acid]	C_19_H_38_O_2_	298.5038		300.09		243.04	201.02		*Rh. adamsii; Rh. sichotense*
15	Kaempferol 5-methyl ether	C_16_H_12_O_6_	300.2629		300.98		283.01; 177.01	264.98	200.98	*Rh. adamsii; Rh. sichotense*
16	Farrerol [5,7-Dihydroxy-2-(4-hydroxyphenyl)-6,8-dimethylchroman-4-one]	C_17_H_16_O_5_	300.3059		301.05		283.04	241.01; 162.96		*Rh. adamsii; Rh. sichotense*
17	Quercetin [2-(3,4-Dihydroxyphenyl)-3,5,7-trihy- droxy-4-*H*-chromen-4-one]	C_15_H_12_O_7_	302.2357	301.09	303.08		285.01; 163.02	180.97; 145.00	162.98	*Rh. adamsii; Rh. sichotense*
18	Dihydroquercetin [Taxifolin; Taxifoliol]	C_15_H_12_O_7_	304.2516	303.09			285.04	266.96; 241.09; 215.05; 135.05	171.02	*Rh. adamsii*
19	Cannabigerorcinic acid [Cannabigerorcinolic acid; Cannabiorcogerolic acid]	C_18_H_24_O_4_	304.3808	303.08			285.05	241.07; 159.07	159.01	*Rh. adamsii*
20	Docosane	C_22_H_46_	310.6006		311.10		293.06; 167.01	259.03	240.97; 162.96	*Rh. adamsii*
21	8-Demethyleucalyptin [5-Hydroxy-4′,7-dimetoxy-6-methylflavone; Pabalate; Sodium salicylate]	C_18_H_16_O_5_	312.3166	311.14			311.10; 182.99			*Rh. adamsii*
22	Arachic acid [Arachidic acid; eicosanoic acid]	C_20_H_40_O_2_	312.5304	311.14		335.04	303.06; 195.01	284.99; 238.14; 163.00	180.89; 135.14	*Rh. adamsii*
23	Azaleatin [5-*O*-Methylquercetin]	C_16_H_12_O_7_	316.2623	315.08			297.01; 167.04	235.04; 149.00		*Rh. adamsii*
24	Myricetin [3,5,7-Trihydroxy-2-(3,4,5-Trihydroxyphenyl)-4*H*-Chromen-4-One]	C_15_H_10_O_8_	318.2351	317.08			299.01; 241.01	240.06; 197.09	238.99; 197.04	*Rh. adamsii; Rh. sichotense*
25	Gossypetin [Articulatidin; Equisporol]	C_15_H_10_O_8_	318.2351		319.07		287.09; 176.98	146.99		*Rh. adamsii*
26	Ampelopsin [Dihydromyricetin; Ampeloptin]	C_15_H_12_O_8_	320.251	319.08			317.01; 275.09	257.12; 217.11		*Rh. adamsii*
27	Heneicosanoic acid [Heneicosylic acid]	C_21_H_42_O_2_	326.557	325.11	327.08		271.01; 217.03; 177.06	149.10		*Rh. adamsii; Rh. sichotense*
28	Myricetin 5-Methyl ether [5-*O*-Methylmyricetin]	C_16_H_12_O_8_	332.2617	331.03			168.94	149.96		*Rh. sichotense*
29	Esculin [Aesculin; Esculoside; Polichrome]	C_15_H_16_O_9_	340.2821		341.09		281.01; 217.11; 151.06	174.96		*Rh. adamsii; Rh. sichotense*
30	Behenic acid [Docosanoic acid]	C_22_H_44_O_2_	340.5836		341.05		323.10; 243.11; 177.04	159.05		*Rh. adamsii; Rh. sichotense*
31	Pentacosane (*N*-Pentacosane)	C_25_H_52_	352.6854		353.12		270.97; 162.97	180.93	162.96	*Rh. sichotense*
32	Chlorogenic acid	C_16_H_18_O_9_	354.3087		355.09		287.05; 164.02	180.95	163.03	*Rh. adamsii; Rh. sichotense*
33	Scopolin [Scopoloside; Scopoletin-7-glucoside; Murrayin]	C_16_H_18_O_9_	354.3087		355.02		323.00	303.96; 184.89	162.86	*Rh. adamsii; Rh. sichotense*
34	Tricosanoic acid [*N*-Tricosanoic acid]	C_23_H_46_O_2_	354.6101		355.08		322.96; 163.00	180.96	162.96	*Rh. adamsii*
35	Lignoceric acid [Tetracosanoic acid]	C_24_H_48_O_2_	368.6367	367.12	369.08		351.08; 285.02; 218.92; 162.98	163.02	144.97	*Rh. adamsii; Rh. sichotense*
36	Fraxin (Fraxetin-8-*O*-glucoside)	C_16_H_18_O_10_	370.3081		371.08		338.99	320.96; 177.03	224.96	*Rh. adamsii; Rh. sichotense*
37	Daurichromenic acid	C_23_H_30_O_4_	370.4819		371.09		352.98; 287.08; 235.08; 179.02	231.04; 205.05; 162.99	180.93; 144.97	*Rh. adamsii; Rh. sichotense*
38	Pentacosanoic acid [*N*-Pentacosanoic acid]	C_25_H_50_O_2_	382.6633		383.07	405.08	351.04; 287.99	229.04	211.03	*Rh. adamsii; Rh. sichotense*
39	Fraxetin-7-*O*-beta-glucuronide	C_16_H_16_O_11_	384.2916	383.09			365.09; 190.96	266.97; 215.02	170.97	*Rh. adamsii; Rh. sichotense*
40	Beta-Sitosterin [Beta-Sitosterol]	C_29_H_50_O	414.7067		415.04		384.02	369.01	338.00	*Rh. adamsii; Rh. sichotense*
41	Cyanidin-3-alpfa-l-arabinoside	C_20_H_19_O_10_	419.3589	418.51			399.05; 319.02; 194.99	381.068 162.02	337.02; 253.08	*Rh. adamsii*
42	Montanic acid [Octacosanoic acid]	C_28_H_56_O_2_	424.743		425.02		407.00	389.00; 348.98; 298.99; 240.97	333.00; 280.97; 173.02	*Rh. adamsii; Rh. sichotense*
43	Alpha-Amyrin [Viminalol]	C_30_H_50_O	426.7174		427.05		408.27; 308.99; 202.91	389.02; 309.01	373.08; 229.10; 142.80	*Rh. adamsii; Rh. sichotense*
44	Lupeol [Fagarasterol; Clerodol; Monogynol B; Lupenol]	C_30_H_50_O	426.7174		427.04		409.01; 202.99	389.02; 247.99	370.96; 264.80	*Rh. adamsii*
45	Dihydroquercetin 3-arabinofuranoside	C_20_H_16_O_11_	432.3344		433.97		352.95	323.53; 271.96	241.95; 181.87	*Rh. adamsii*
46	Afzelin [ Kaempferol-3-Rhamnoside; Kaempferin]	C_21_H_20_O_10_	432.3775	431.04			413.00; 372.98; 216.94	354.95; 167.01	336.98; 148.91	*Rh. sichotense*
47	Quercetin-3-*O*-beta-xyloside (Reynoutrin; Quercetin 3-*O*-Beta-d-Xylopyranoside)	C_20_H_17_O_11_	433.3424		434.90		302.94	256.92; 164.96	228.91; 159.11	*Rh. sichotense*
48	Avicularin (Quercetin 3-Alpha-l-Arabinofuranoside; Avicularoside)	C_20_H_18_O_11_	434.3503	433.09			415.07; 335.01; 176.98	397.06; 190.99	353.07; 253.99	*Rh. adamsii; Rh. sichotense*
49	Pentoside dihydroquercetin		436	435.16			416.54; 300.99; 231.01	397.02; 205.96	361.11; 283.02; 188.80	*Rh. adamsii; Rh. sichotense*
50	Erithrodiol [3beta-Erytrodiol]	C_30_H_50_O_2_	442.7168	441.12			425.06; 381.05; 300.03; 217.04	363.06; 246.02	319.08; 201.02	*Rh. adamsii*
51	Uvaol	C_30_H_50_O_2_	442.7168		443.22		425.01; 233.07	407.02; 325.01	388.99; 231.11	*Rh. adamsii; Rh. sichotense*
52	Quercitrin [Quercetin 3-l-Rhamnoside; Quercetrin]	C_21_H_20_O_11_	448.3769		448.89		370.95; 282.93	352.95; 176.98	334.90; 222.92; 176.97	*Rh. adamsii*
53	Catechin-7-*O*-glucoside	C_21_H_24_O_11_	452.4087		453.17		435.15; 336.07; 209.06	417.16; 336.11; 226.12	209.09	*Rh. sichotense*
54	Micromeric acid	C_30_H_46_O_3_	454.6844		455.05		408.98; 246.98	391.05; 287.96	250.96	*Rh. adamsii*
55	Hyperoside (Quercetin 3-*O*- galactoside; Hyperin)	C_21_H_20_O_12_	464.3763		465.02		302.91	256.94; 190.87	228.96; 172.75	*Rh. sichotense*
56	Quercetin 3-*O*-glucoside [Isoquercitrin]	C_21_H_20_O_12_	464.3763		465.08		447.00	386.96	369.12; 172	*Rh. sichotense*
57	Alpha.-Tocopherol-Beta-d-Mannoside [Dihydro-2H-Chromen-6-YI Hexofuranoside]	C_35_H_60_O_7_	592.8467		593.11		533.08	461.10	433.11	*Rh. sichotense*

## Supplementary Materials

The following are available online, Figure S1: Chemical profiles of the *Rh. adamsii* sample represented ion chromatogram from SC-CO_2_ extract; Figure S2: Chemical profiles of the *Rh. sichotense* sample represented ion chromatogram from SC-CO_2_ extract.

## References

[1] Pojarkova A.I. (1952). Genus *Ericaceae,* D.K.-vacciniaceous. Flora USSR.

[2] Aleksandrova M.S. (1975). Rhododendrons of Natural Flora of the USSR.

[3] Zaytseva G.Y., Ambros E.V., Karakulov A.V., Novikova T.I. (2018). Flow cytometric determination of genome size and ploidy level of some frost-resistant cultivars and species of Rhododendron L. native to Asian Russia. Botanica Pacifica. Bot Pac..

[4] Belousova N.I., Khan V.A., Tkachev A.V. (1999). The chemical composition of essential oil of Rhododendron. Khimiya Rastitel’nogo Syr′iya (Chem. Plant Raw Mater.).

[5] Belousov M.V., Komissarenko N.F., Berezovskaya T.P., Tochkova T.V. (1994). Content of flavonoids and coumarins in the Siberian—Far Eastern species of the *Ericaceae* family. Rastit. Resur..

[6] Varlygina T.I., Kamelin R.V., Kiseleva K.V. (2008). Red Data Book of Russian Federation.

[7] Firsov G.A., Egorov A.A., Byalt V.V., Neverovsky V.J., Orlova L.V., Volchanskaya A.V., Lavrentyev N.V. (2010). Arboreal plants of the Red Data Book of Russia in collection of Saint Petersburg Forest-Technical Academy. Hortus Botanicus..

[8] Khokhryakov A.P., Mazurenko M.T. (1991). Vascular plants of the Soviet Far East. Science.

[9] Hubich A.I., Puchkova K.V., Zalesskaya N.A., Kryuchkova N.V. (2018). The investigation of the adaptogenic properties of Rhododendron adamsii Rehder. on experimental models in vivo. J. Belarus. State Univ. Biol..

[10] Mirovich V.M., Konenkina T.A., Fedoseeva G.M. (2008). Qualitative structure od essential oil of *Rhododendron adamsii* and *parvifolium*, growing in East Siberia. Siberian Med. J..

[11] Belousova N.I., Khan V.A. (1990). Bicyclic monoterpenoids of the essential oil of *Ledum palustre*. Chem. Nat. Compd..

[12] Kurshakova G.V., Fedorov A.A., Yakimov P.A. (1961). Some data on the chemical composition and pharmacological effect of rhododendron Adams–*Rhododendron adamsii* Rend. Trudy Botanicheskogo instituta im. V. L. Komarova AN SSSR.

[13] Belousov M.V., Berezovskaya T.P., Komissarenko N.F., Tikhonova L.A. (1998). Flavonoids of Siberian and Far-Eastern species of rhododendrons of the subsgenus Rhodorastrum. Chem. Nat. Compd..

[14] Fini A., Brunetti C., Di Ferdinando M., Ferrini F., Tattini M. (2011). Stress-induced flavonoid biosynthesis and the antioxidant machinery of plants. Plant. Signal. Behav..

[15] Rogachev A.D. (2009). Phytochemical study of Rhododendron Adamsii Rheder. Ph.D.’s Thesis.

[16] Rogachev A.D., Fomenko V.V., Sal’nikova O.I., Pokrovskii L.M., Salakhutdinov N.F. (2006). Comparative analysis of essential oil compositions from leaves and stems of *Rhododendron adamsii, R. aureum*, and *R. dauricum*. Chem. Nat. Compd..

[17] Razgonova M.P., Zacharenko A.M., Kalenik T.K., Nosyrev A.E., Stratidakis A.K., Mezhuev Y.O., Burykina T.I., Nicolae A.C., Arsene A.L., Tsatsakis A.M. (2019). Supercritical fluid technology and supercritical fluid chromatography for application in ginseng extracts. Farmacia.

[18] Razgonova M., Zakharenko A., Shin T.-S., Chung G., Golokhvast K. (2020). Supercritical CO_2_ Extraction and Identification of Ginsenosides in Russian and North Korean Ginseng by HPLC with Tandem Mass Spectrometry. Molecules.

[19] Morozov Y.A., Pupykina K.A., Blagorazumnaya N.V., Aliev A.M., Morozova E.V. (2018). Comparative analysis of carbon dioxide extracts from plant material of Schisandra chinensis: Leaves, woody stems, rhizomes with roots. Med. Bull. Bashkortostan..

[20] Aliev A.M., Radjabov G.K., Musaev A.M. (2015). Dynamics of supercritical extraction of biological active substances from the *Juniperus communis* var. *saxatillis*. J. Supercrit. Fluids.

[21] Rovetto L.J., Aieta N.V. (2017). Supercritical carbon dioxide extraction of cannabinoids from *Cannabis sativa* L.. J. Supercrit. Fluids.

[22] Baldino L., Della Porta G., Sesti Osseo L., Reverchon E., Adami R. (2018). Concentrated oleuropein powder from olive leaves using alcoholic extraction and supercritical CO_2_ assisted extraction. J. Supercrit. Fluids..

[23] Mehariya S., Iovine A., Di Sanzo G., Larocca V., Martino M., Leone G.P., Casella P., Karatza D., Marino T., Musmarra D. (2019). Supercritical fluid extraction of lutein from *Scenedesmus almeriensis*. Molecules.

[24] Leone G.P., Balducchi R., Mehariya S., Martino M., Larocca V., Di Sanzo G., Iovine A., Casella P., Marino T., Karatza D. (2019). Selective Extraction of ω-3 Fatty Acids from *Nannochloropsis* sp. Using Supercritical CO_2_ extraction. Molecules.

[25] Senica M., Stampar F., Miculic-Petkovsek M. (2019). Different extraction processes affect the metabolites in blue honeysuckle (*Lonicera caerulea* L. subsp. *edulis*) food products. Turk. J. Agric. For..

[26] Colak A.M., Okatan V., Polat M., Guclu S.F. (2019). Different harvest times affect market quality of *Lycium barbarum* L. berries. Turk. J. Agric. For..

[27] Baldino L., Reverchon E. (2018). Challenges in the production of pharmaceutical and food related compounds by SC-CO_2_ processing of vegetable matter. J. Supercrit. Fluids.

[28] Popova A.S., Ivahnov A.D., Skrebets T.E., Bogolitsyn K.G. (2018). Supercritical fluid extraction of carotenoids and chlorophyll from *Ledum palustre*. Khimiya Rastitel’nogo Syr’iya (Chem. Veg. Raw Mater.).

[29] Baananou S., Bagdonaite E., Marongiu B., Piras A., Porcedda S., Falconieri D., Boughattas N.A. (2015). Supercritical CO_2_ extract and essential oil of aerial part of *Ledum palustre* L.—Chemical composition and anti-inflammatory activity. Nat. Prod. Res..

[30] Bukreyeva T.V., Shavarda A.L., Matusevich O.V., Morozov M.A. (2013). Ursane, oleanane, lupine triterpenoids from leaves of *Ledum palustre* (Ericaceae) from North-West Russia. Rastit. Resur..

[31] Butkiene R., Sakociute V., Latvenaite D., Mockute D. (2008). Composition of young and aged shoot essential oils of the wild *Ledum palustre* L.. Chemija.

[32] Buzuk A.G., Buzuk G.N. (2016). The study of chemical variability of essential oil composition of *Ledum palustre* L., growing on the territory of the republic of Belarus. Vestnik Farmacii..

[33] Dampc A., Luczkiewicz M. (2013). *Rhododendron tomentosum (Ledum palustre)*. A Review of traditional use based on current research. Fitoterapia.

[34] Dede E., Genc N., Elmastas M., Aksit H., Erenler R. (2019). Chemical constituents Isolated from *Rhododendron ungernii* with Antioxidant Profile. Nat. Prod. J..

[35] Ganina M.M. (2015). and Popova, O.I. Content of phenolic compounds in shoots of *Ledum* procumbent (*Ledum decumbens* Lodd. ex Steud) growing on the territory of the Yamalo-nenets autonomous district. Khim.-Farm. Zh..

[36] Han F., Li Y., Ma L., Liu T., Wu Y., Hu R., Song A., Yin R. (2016). A rapid and sensitive UHPLC-FT-ICR MS/MS method for identification of chemical constituents in *Rhodiola crenulata* extract, rat plasma and rat brain after oral administration. Talanta.

[37] Harborne J.B., Williams C.A. (1971). Leaf survey of flavonoids and simple phenols in the genus Rhododendron. Phytochemistry.

[38] Izotov D.V., Tagiltsev Y.G., Kolesnikova R.D., Tsyupko V.A. (2010). Biologically active substances of Far-Eastern Labrador tea. Lesnoy J..

[39] Karpova E.A., Karakulov A.V. (2013). Flavonoids of some Rhododendron species of flora of Siberia and the Far East. Khimiya Rastitel’nogo Syr’ya (Chem. Plant Raw Mater.).

[40] Karakulov A.V., Karpova E.A., Vasiliev V.G. (2018). Ecological and geographical variation of morphometric parameters and flavonoid composition of *Rhododendron parvifolium*. Turczaninowia.

[41] Korotaeva M.S., Belousov M.V., Fursa N.S. (2008). Flavonoids and hydroxycinnamic acids content in *Ledum palustre (Ericaceae)* above-ground part. Rastit. Resur..

[42] Mirovich V.M., Fedoseeva G.M., Zjubr T.P., Fedoseev A.P., Paisova O.I., Kuklina L.B. (2006). Elaboration of the method of receipt of the dry extract from sprouts of *Rhododendron adamsii*, having actoprotective and antimicrobic activity. Sibirskii medicinskii Zhurnal..

[43] Pandey R., Kumar B. (2016). HPLC-OTOF-MS/MS-based rapid screening of phenolics and triterpenic acids in leaf extracts of *Ocimum* species and heir interspecies variation. J. Liq. Chromatogr. Relat. Technol..

[44] Plyashechnik M.A. (2012). Chemical composition of *Ledum palustre* L. essential oil under increasing nitrogen availability in soils of cryolitzone (Central Evenkia). Khimiya Rastitel’nogo Syr’iya (Chem. Plant Raw Mater.).

[45] Raal A., Orav A., Gretchushnikova T. (2014). Composition of the essential oil of the *Rhododendron tomentosum* Harmaja from Estonia. Nat. Prod. Res..

[46] Suzuki H., Sasaki R., Ogata Y., Nakamura Y., Sakurai N., Kitajima M., Takayama H., Kanaya S., Aoki K., Shibata D. (2008). Metabolic profiling of flavonoids in Lotus japonicus using liquid chromatography Fourier transform ion cyclotron resonance mass spectrometry. Phytochemistry.

[47] Taamalli A., Arráez-Román D., Abaza L., Iswaldi I., Fernández-Gutiérrez A., Zarrouk M., Segura-Carretero A. (2015). LC-MS-based metabolite profiling of methanolic extracts from the medicinal and aromatic species Mentha pulegium and Origanum majorana. Phytochem. Anal..

[48] Ul’yanovskii N.V., Kosyakov D.S., Pokryshkin S.A., Bogolitsyn K.G., Ul’yanovskaya O.S. (2014). Study of volatile compounds composition of *Ledum palustre* L. using the method of thermodesorption gas chromatography -mass spectrometry. Khimiya Rastitel’nogo Syr’iya (Chem. Plant Raw Mater.).

[49] Yang S.T., Wu X., Rui W., Guo J., Feng Y.F. (2015). UPLC/Q-TOF-MS analysis for identification of hydrophilic phenolics and lipophilic diterpenoids from Radix *Salviae Miltiorrhizae*. Acta Pharm..

[50] Zaytseva N.V., Pogulyaeva I.A. (2014). Chromatographic Analysis of Chemical Composition of the Genus Rhododendron Plants Growing on the Mountain of Evota (South Yakutia). J. Chem. Chem. Eng..

[51] Jin C., Strembiski W., Kulchytska Y., Micetich R.G., Daneshtalab M. (1999). Flavonoid glycosides from *Ledum palustre* L. subsp. *decumbens* (Ait.) Hulton. DARU. J. Pharm. Sci..

[52] Komarova N.I., Rogachev A.D., Chernyak E.I., Morozov S.V., Fomenko V.V., Salakhutdinov N.F. (2009). Quantitative HPLC determination of main flavonoid content of *Rhododendron adamsii* leaves and stems. Chem. Nat. Compd..

[53] Okhlopkova Z.M., Chirikova N.K. (2012). Component composition analysis of essential oil of the *Ledum palustre* L., growing in Yakutia. Fundam. Res..

[54] (2016). Russian State Pharmacopeia XIII. http://pharmacopoeia.ru/en/gosudarstvennaya-farmakopeya-xiii-online-gf-13-online/.

