# Supplementary figures and images for: Comparative Analysis of Far East Sikhotinsky Rhododendron (Rh. sichotense) and East Siberian Rhododendron (Rh. adamsii) Using Supercritical CO2-Extraction and HPLC-ESI-MS/MS Spectrometry
Source: Molecules. 2020 Aug 19;25(17):3774. doi: 10.3390/molecules25173774 (PMC7503641; doi:10.3390/molecules25173774)

# Supplementary

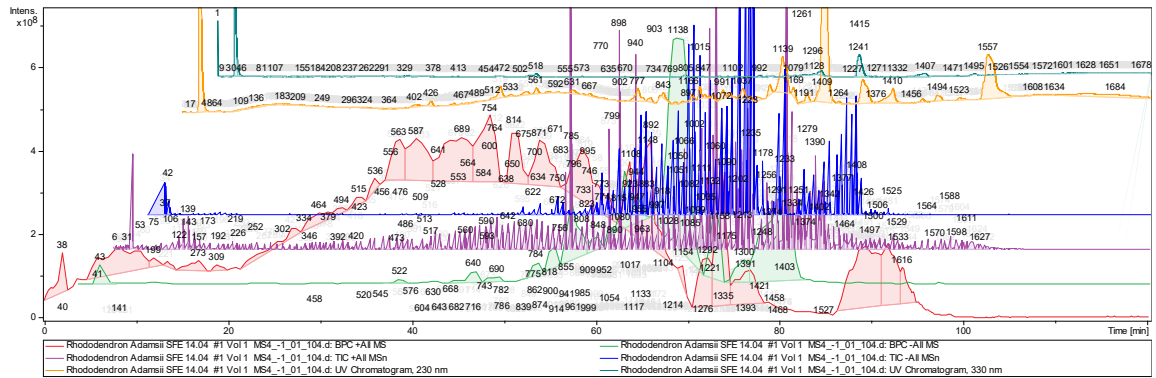

Supplement: Supplementary file 1 [file molecules-25-03774-s001.pdf]
